# Proteoglycans and Glycosaminoglycans in Stem Cell Homeostasis and Bone Tissue Regeneration

**DOI:** 10.3389/fcell.2021.760532

**Published:** 2021-11-30

**Authors:** Jiawen Chen, Tianyu Sun, Yan You, Buling Wu, Xiaofang Wang, Jingyi Wu

**Affiliations:** ^1^ School of Stomatology, Southern Medical University, Guangzhou, China; ^2^ Department of Periodontology, Stomatological Hospital, Southern Medical University, Guangzhou, China; ^3^ Department of Endodontics, Shenzhen Stomatology Hospital, Southern Medical University, Shenzhen, China; ^4^ Department of Biomedical Sciences, Texas A&M University College of Dentistry, Dallas, TX, United states; ^5^ Center of Oral Implantology, Stomatological Hospital, Southern Medical University, Guangzhou, China

**Keywords:** proteoglycan, glycosaminoglyans, mesenchymal stem cell, stem cell homeostasis, differentiation, self-renewal, tissue engineering, osteogenesis

## Abstract

Stem cells maintain a subtle balance between self-renewal and differentiation under the regulatory network supported by both intracellular and extracellular components. Proteoglycans are large glycoproteins present abundantly on the cell surface and in the extracellular matrix where they play pivotal roles in facilitating signaling transduction and maintaining stem cell homeostasis. In this review, we outline distinct proteoglycans profiles and their functions in the regulation of stem cell homeostasis, as well as recent progress and prospects of utilizing proteoglycans/glycosaminoglycans as a novel glycomics carrier or bio-active molecules in bone regeneration.

## Introduction

Proteoglycans are large glycoproteins that are expressed abundantly on the cell surface and in the extracellular matrix (ECM) with critical structural and functional roles in tissue development and in the regulation of various physiological processes. Proteoglycans act as liaisons between the intracellular and extracellular space by regulating multiple signals (Xie and Li, 2019). As a consequence, proteoglycans are involved in various physiological processes, such as tissue morphogenesis (Nakato and Li, 2016), stem cell homeostasis (Kraushaar et al., 2013; Izumikawa et al., 2014), and the regulation of cellular growth and differentiation (Chen et al., 2007). The expression patterns of proteoglycans hold temporal and spatial specificity to constantly adapt to multiple biological environments. The rapid development of glycomic and glycoproteomic analytical approaches make it possible to mediate the homeostasis of stem cells by determining and exploiting their functional fragments (Sebastião et al., 2021). In addition, much attention has focused on utilizing bio-synthesized functional glycosaminoglycan chains (GAGs) that decorate proteoglycans to synthesize novel biomaterials and scaffolds for tissue regeneration. In this review, we summarize the structural features and the roles of proteoglycan and GAGs in the regulation of stem cell homeostasis and outline the application and prospects of proteoglycan/GAGs-derived biomaterials in bone regeneration.

## Structural and Functional Characteristics of Proteoglycans and Glycosaminoglycans

Proteoglycans are a unique class of glycoproteins consisting of a core protein to which one or more GAGs are covalently attached. They are ubiquitously expressed on cell surfaces and throughout the ECM of eukaryotic cells (Iozzo and Schaefer, 2015). The expression of proteoglycans present spatiotemporal features (Gao et al., 2018; Wu et al., 2020a) during different biological and pathological processes, such as stem cell homeostasis (Smith et al., 2011; Mikami and Kitagawa, 2017; Wang et al., 2017; Yasa et al., 2017), development of tissues and organs (Gualeni et al., 2013), and cancer initiation and progression (de Wit et al., 2017; Nagarajan et al., 2018).

**FIGURE 1:**
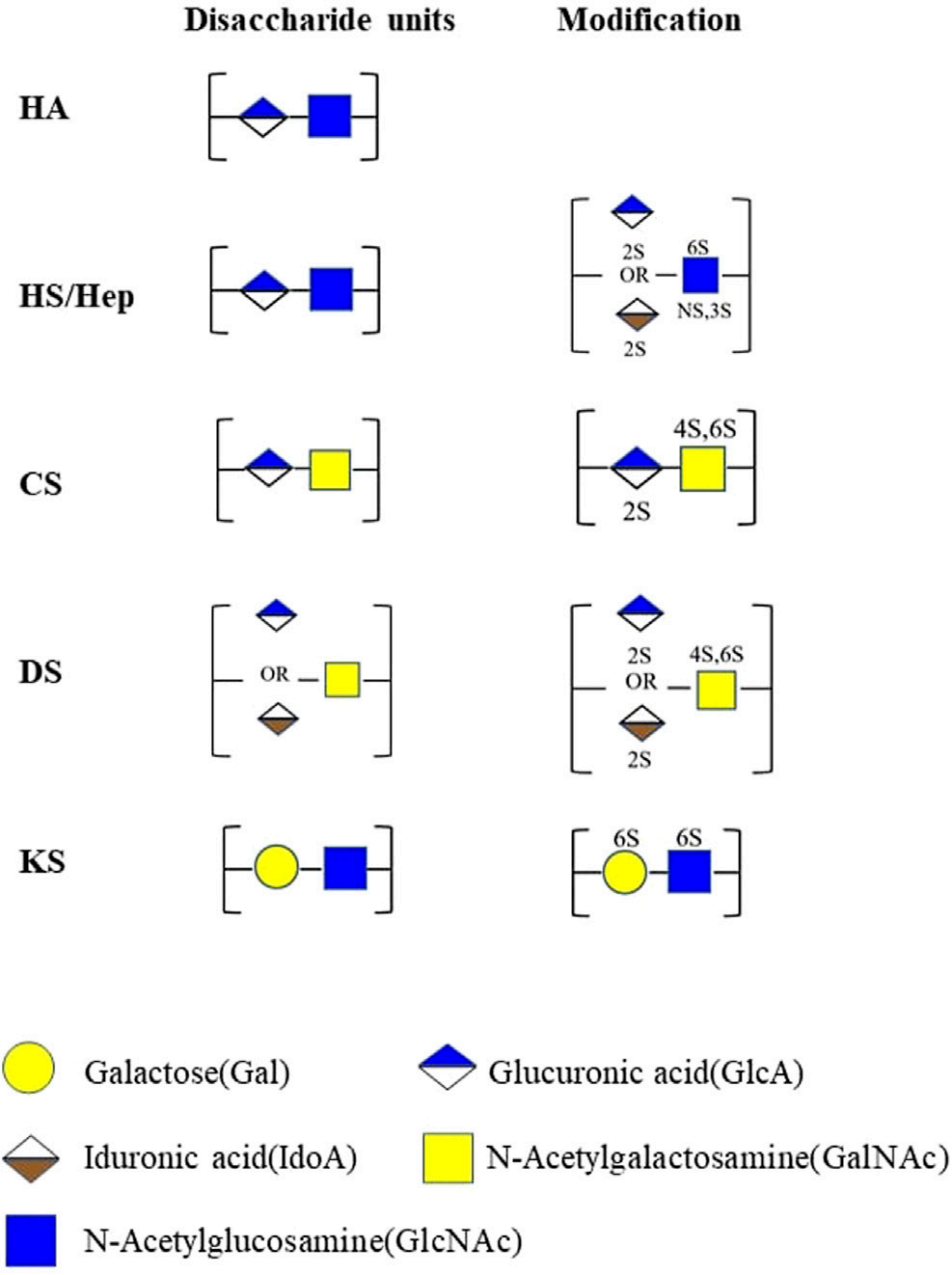
Structures of GAGs and their modification. HA, hyaluronan; HS, heparan sulfate; Hep, heparin; CS, chondroitin sulfate; DS, dermatan sulfate; KS, keratan sulfate; 2S, 2-O-sulfation; 6S, 6-O-sulfation; 3S, 3-O-sulfation; 4S, 4-O-sulfation; NS, N-sulfated glucosamine. Monosaccharides in this figure are represented in accordance with the symbol nomenclature for glycans (SNFG) (Varki et al., 2015).

**FIGURE 2:**
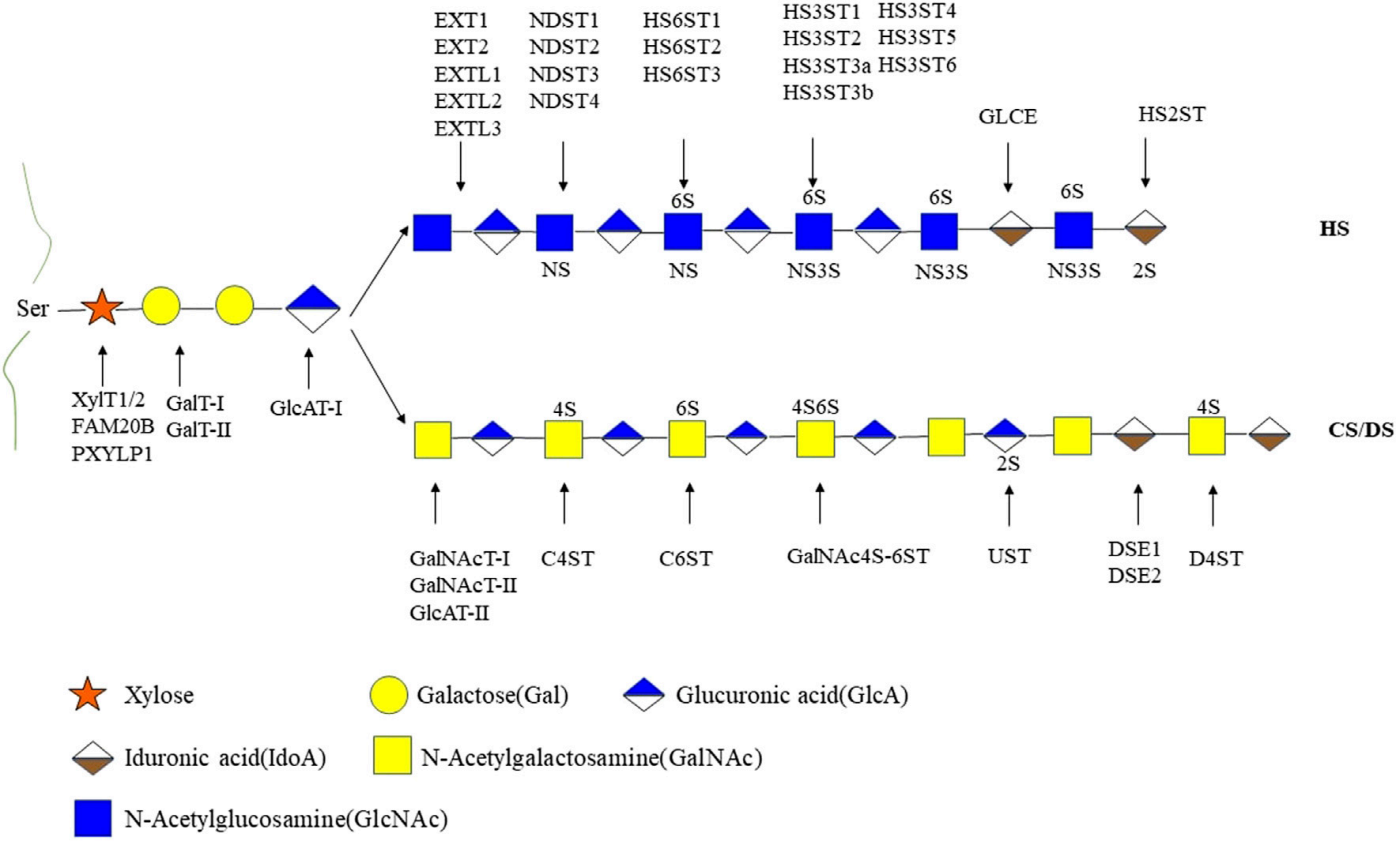
The biosynthetic pathways of heparan sulfate and chondroitin sulfate/dermatan sulfate. HS, heparan sulfate; CS, chondroitin sulfate; DS, dermatan sulfate; 2S, 2-O-sulfation; 6S, 6-O-sulfation; 3S, 3-O-sulfation; 4S, 4-O-sulfation; NS, N-sulfated glucosamine. XylT1/2, xylosyltransferase 1/2; FAM20B, family with sequence similarity member 20-B; PXYLP1, 2-phosphoxylose phosphatase; GalT-I, galactosyltransferase-I; GalT-II, galactosyltransferase-II; GlcAT-Ⅰ, glucuronyltransferase-Ⅰ; EXT1, exostosin glycosyltransferase 1; EXT2, exostosin glycosyltransferase 2; EXTL1, exostosin like glycosyltransferase 1; EXTL2, exostosin like glycosyltransferase 2; EXTL3, exostosin like glycosyltransferase 3; NDST1, N-sulfotransferase; NDST2, N-sulfotransferase 2; NDST3, N-sulfotransferase 3; NDST4, N-sulfotransferase 4; HS6ST1, heparan sulfate 6-O-sulfotransferase 1; HS6ST2, heparan sulfate 6-O-sulfotransferase 2; HS6ST3, heparan sulfate 6-O-sulfotransferase 3; HS3ST1, heparan sulfate 3-O-sulfotransferase 1; HS3ST2, heparan sulfate 3-O-sulfotransferase 2; HS3ST3a, heparan sulfate 3-O-sulfotransferase 3a; HS3ST3b, heparan sulfate 3-O-sulfotransferase 3b; HS3ST4, heparan sulfate 3-O-sulfotransferase 4; HS3ST5, heparan sulfate 3-O-sulfotransferase 5; HS3ST6, heparan sulfate 3-O-sulfotransferase 6; GLCE, C-5 epimerase; HS2ST, heparan sulfate 2-O-sulfotransferase; GalNAcT- I, GalNAc transferase-I; GalNAcT- IIs, GalNAc transferase-II; GlcAT- II,β1,3-glucuronyltransferase- II; C4ST, chondroitin 4-O-sulfotransferase; C6ST,; GalNAc4S-6ST,; UST, uronyl 2-O-sulfotransferase; DSE1, DS epimerase1; DSE2, DS epimerase2; D4ST, dermatan 4-O-sulfotransferase; GalNAc4S-6ST, GalNAc 4-sulfate 6-O-sulfotransferase. Monosaccharides in this figure are represented in accordance with the symbol nomenclature for glycans (SNFG) (Varki et al., 2015).

GAGs are negatively charged unbranched polysaccharides with repeating disaccharides. The composition of GAG chains determines the biological function associated with proteoglycans (Mikami and Kitagawa, 2017). Different types of sulfated glycosaminoglycan are covalently attached to their core proteins by identical linkages via an O-link to serine residues. Based on the different patterns of their repeating disaccharide, GAGs can be divided into heparan sulfate (HS), chondroitin sulfate (CS), dermatan sulfate (DS), keratan sulfate (KS), hyaluronic acid (HA), and heparin (HEP). Among these types of GAGs, HA is the only one with a linear and unbranched backbone consisting of a repeating disaccharide unit composed of glucuronic acid (GlcA) and N-acetyl-D-glucosamine (GlcNAc) without any sulfate groups (Figure 1). GAGs undergo extensive modifications by sulfotransferase and endosulfatase, which gave rise to various sulfation patterns. HS are linear polysaccharides composed of GlcA-GlcNAc repeating units and modified by epimerization (C5-epimerase), sulfation (N-,2-O-,3-O-,6-O-sulfotransferases), and by desulfation (endosulfatase). Similarly, CS/DS chains are subjected to marked structural modification by sulfation and epimerization of the repeating GalNAc-GlcA disaccharide units. These modifications result in GAGs with high heterogeneity in terms of chain length and size, sulfation patterns and degrees (Karamanos et al., 2018) (Figures 1, 2).

In comparison with HS and CS/DS, the repeating disaccharide units of KS are composed of Gal and GlcNAc (Figure 1). Based on the different structures in the linkage oligosaccharides, KS can be further divided in to KS-I, KS-II, and KS-III (Caterson and Melrose, 2018). The N-linked KS-I is predominantly expressed in the cornea where it displays variable degrees of sulfation ranging from non-sulfated polylactosamine, mono-sulfated, and disulfated disaccharide regions. O-linked KS-II is highly expressed in cartilage, with a higher degree of sulfation and disulfated disaccharides interrupted by occasional mono-sulfated N-acetyllactosamine residues. KS-III is synthesized by the extension of O-linked mannose and is mainly expressed in the brain and is highly sulfated.

**FIGURE 3:**
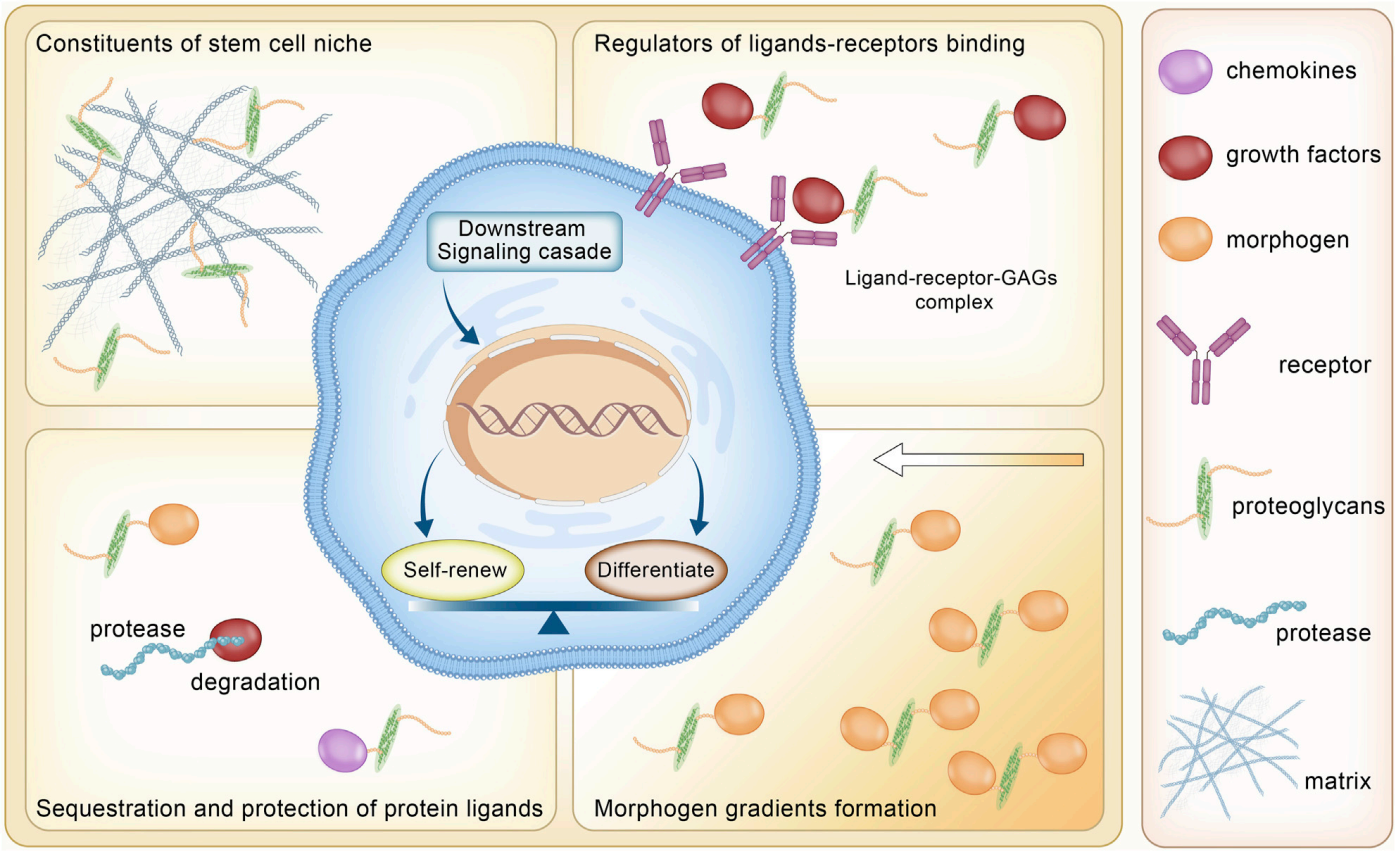
Roles of proteoglycans in stem cell homeostasis.

Proteoglycans not only act as a matrix framework but also regulate various signaling cascades governing biological process. Proteoglycans and glycosaminoglycans have been involved in multiple cellular signaling pathways (Figure 3). They help shape the protein diffusion gradient in the ECM through interactions with various protein ligands. During embryogenesis, proteoglycans limit the diffusion of extracellular ligands such as Wnt proteins (Mii and Takada, 2020) and fibroblast growth factor (FGF) (Ornitz and Marie, 2015; Balasubramanian and Zhang, 2016) from freely interacting with their receptors. In other conditions, proteoglycans also serve as co-receptors for growth factors, facilitating signal transmission. Heparan sulfate proteoglycans (HSPGs) on the cell surface form a large complex with fibroblast growth factor 2 (FGF2) and fibroblast growth factor receptor 1 (FGFR1) in the mediation of the FGF2/FGFR/extracellular signal-regulated kinase 1/2 (ERK1/2) signaling pathway (Ornitz, 2000). In addition, proteoglycans also protect growth factors, cytokines, and chemokines from proteolysis by binding to them.

Interestingly, both the core protein and GAG units contribute to the biological activity of proteoglycans. GAGs are widely studied due to their structural heterogeneity, which endows them with potential diversity in modulation of biological processes. Defects in GAGs biosynthesis have been associated with human congenital diseases and disorders such as skeletal and connective tissue dysplasia (Soares da Costa et al., 2017) and disturbed embryonic development in several animal models (Vogel et al., 2012). Currently, genetic modifications have been the most popular and useful tool for elucidating the functions of GAGs in mediating stem cell development and differentiation. *Ext-1* ^*−/−*^ ESCs and *GlcAT-1* ^*−/−*^ ESCs failed to differentiate and maintained pluripotency, indicating that HS and CS/DS play an essential role in mediating stem cell lineage commitment (Kraushaar et al., 2010; Izumikawa et al., 2014). Attenuated differentiation was also observed in *Ndst1/2* ^*−/−*^ ESCs which lacked the N-sulfation of HS (Lanner et al., 2010; Forsberg et al., 2012). Although genetic modifications helped to unveil the roles of GAGs to some extent, synergistic effects and compensation mechanisms between different proteoglycans and GAGs suggested a higher complexity of GAGs-mediated stem cell behaviors (Corsi et al., 2002; Wadhwa et al., 2007; Bachvarova et al., 2020). To better decipher the specific sulfation pattern of proteoglycans, chemical, and enzymatic synthesis of GAGs (DeAngelis et al., 2013; Gottschalk and Elling, 2021) with well-defined structures as well as characterization techniques such as nuclear magnetic resonance spectroscopy (NMR), surface plasmon resonance (SPR), and chromatography have been utilized to better understanding the binding interactions between protein ligands and GAGs (Li et al., 2016; Pomin and Wang, 2018; Vallet et al., 2021). These chemoenzymatically synthesized GAGs have mainly been used for exogenous addition to cellular and animal models with the objective to determining how GAGs interact with growth factors *in vitro* and *in vivo*. Linhardt and his group have developed a rapid and convenient method that utilized heparin chip and SPR to reveal the binding kinetics and affinities of hep with different growth factors including FGFs, HFG and TGF β-1 *in vitro*. This method also allows efficient exploration of GAGs chain-size dependence and the effect of heparin sulfate group in growth factor interaction (Zhang et al., 2009a; Zhang et al., 2019).

By systematic knockout and/or knock-in of the genes encoding the enzymes in proteoglycans biosynthesis, researchers have successfully constructed cell libraries presenting distinct glycosaminoglycans with a broad repertoire of modifications (Chen et al., 2018; Qiu et al., 2018). This has enabled the exploration of the structure-function relationships of GAGs in cell lines. Through mutant cell libraries of mouse lung endothelial cells (MLEC) and Chinese hamster ovary cells (CHO), Qiu et al. (2018) and Chen et al. (2018) determined that N-sulfation and 2-O-sulfation of HS are critical for FGF2 binding and that the overall sulfation level was more important than the sulfate pattern for FGF2-FGFR1 signaling.

By assembling specifically labeled GAGs on proteoglycans in living cells, Wang et al. (2020) revealed the irreplaceable role of HS in the formation of the glypican-3-frizzeld-7 complex, which plays a vital in regulating Wnt signaling. Collectively, the structural and compositional features of GAGs greatly influence the biological functions of proteoglycans and their ability to mediate signaling transduction. However, cell-based libraries only provide information using specific cell-types such as immortalized CHO cells and MLEC. Whether such structure-relationships also apply to other mammalian cells remains unclear. To provide a more comprehensive and systematic view of GAGs-mediated regulation of signaling transduction, further studies should be undertaken to investigate the structure–function relationship of glycosaminoglycans in more physiologically relevant cells.

## Distinct Proteoglycan Expression Patterns During Stem Cells Development

Stem cells are characterized by their unique ability to self-renew and differentiate into specialized cells (Yamanaka, 2020). There are various sources of stem cells with varying potencies. Pluripotent cells are embryonic stem cells (ESCs) derived from the inner cell mass of embryos and induced pluripotent cells are formed by reprogramming of somatic cells. They can differentiate and form tissues of all three germ layers. In addition, multipotent stem cells are characterized by less potency compared to ESCs as they are derived from a single germ layer, such as mesenchymal stem cells (MSCs), which can differentiate along adipose, bone, and cartilage lineages. In addition, oligopotent stem cells exist in different tissues to form terminally differentiated cells of a specific tissue (Vats et al., 2005). The homeostasis of stem cells requires tight regulation by a precise intra/extracellular signaling network. Accumulated evidence has demonstrated that intracellular mediators such as growth factors, morphogens, non-coding RNAs, and transcription factors are involved in the controlled equilibrium between quiescence and activation of stem cells (Sarkar and Hochedlinger, 2013; Garg et al., 2017; Fico et al., 2019). Furthermore, extracellular factors including signaling molecules, metabolites, environmental cues active in the stem cell niche also contribute to the maintenance of stem cell homeostasis (Morrison and Spradling, 2008; Ryall et al., 2015). Moreover, the molecular mechanisms that govern the self-renewal and differentiation of stem cell regulation is highly cell-specific. The spatial and temporal context of a stem cell greatly determines how the cell interprets the messages of signaling molecules and the potentiation of downstream signal transduction (Kléber and Sommer, 2004; Varga and Wrana, 2005; Watabe and Miyazono, 2009; Semba et al., 2020). Thus, substantial researches have demonstrated a complex regulatory network of stem cell homeostasis and is highly dependent on the spatio and temporal context. Despite the significant advances in stem cell biology, relatively little is known about the biological role of proteoglycans that are present within the stem cell niche and serve as crucial mediators for transmitting extracellular cues into the intracellular responses.

A comprehensive insight into the biological functions of proteoglycans in stem cell homeostasis and differentiation will provide essential knowledge to better understand the dynamics of cell fate specifications, and to establish improved stem cell-based medical applications.

### Embryonic Stem Cells

ESCs are pluripotent stem cells characterized by indefinite proliferation and potential for differentiation into the cell types constituted by the three embryonic germ layers, providing an unlimited supply of cell resources for tissue regeneration *in vitro* (Yamanaka, 2020). When removed from feeder layers and transferred to suspension cultures, ESCs begin to differentiate into multicellular aggregates, termed embryoid bodies (EBs), which will later differentiate into three embryonic germ layers and give rise to terminally differentiated cells such as osteoblasts and hepatocytes under specific culture conditions (Murry and Keller, 2008).

Understanding the expression patterns of GAGs and core proteins is the first step in determining how these might function during stem cell differentiation. Narin et al. (2007) observed significant content and composition changes of both core proteins and GAGs during differentiation of ESCs. The authors reported that during the initial differentiation into EBs, the expression levels of proteoglycans core proteins increased (by > 10-fold) in 39% remained unchanged in 56%, and decreased in 6% of the core proteins detected. Further, the most striking increase was observed in glypican-5 (by 100-fold) (Nairn et al., 2007), a type of HSPG located on the cell surface, which have recently found to bind and sequester Shh in the ECM to form a graded distribution of Hh essential for embryogenesis (Guo and Roelink, 2019, 5). Apart from core proteins, HA presented a 13-fold and 24-fold upregulation during ESC differentiation into the extraembryonic ectoderm (ExE) and EBs. CS/DS synthesis was elevated by 6-fold, 4-fold higher in the ExE and EBs. HS synthesized by the ExE and EBs was 5-fold and 8-fold higher respectively, compared to ESCs (Nairn et al., 2007). In addition, GAGs sulfation also undergoes strict regulation during ESCs differentiation. Sulfotransferases such as NDSTs, HS6ST, HS3ST, UST, CHST11, CHST12, and D4ST1 were upregulated, which is suggestive of increased synthesis of highly sulfated GAGs such as CS-E, highly sulfated HS, and enhanced 2-sulfation of both CS/DS and HS during ESCs differentiation (Nairn et al., 2007). Similarly, Fujitani et al. (2013) also confirmed the positive role of GAGs sulfation during stem cell differentiation. Their results demonstrated that hESCs and hiPSCs produced significantly more nonsulfated/low-sulfated GAGs including nonsulfated chondroitin (CS-0S), nonsulfated heparan (HS-0S), 2- and/or 4-sulfated chondroitin (CS-2S4S, CS-2S, and CS-4S), and N- or 6-sulfated heparan (HS-6S and HS-NS) than in non-stem cells.

In the neural differentiation of ESCs towards Sox1^+^ neural progenitor cells (NPCs), a comparison of the disaccharide composition of HS from ESCs and NPCs showed a dramatic increase in the amounts in N-sulfation, 6-O-sulfation, and 2-O-sulfation which was in good agreement with the significant increased mRNA levels of *NDST4* (by 9000-fold), *NDST3* (by 18-fold), *6OST-2* (by2.7-fold) and *6OST-3* (by 8-fold) expression (Johnson et al., 2007).

GAGs profile is highly dynamic during stem cell differentiation as demonstrated by previous researches. As the roles of GAGs and how it affects stem cell behaviors are also cell-type specific. Whether GAGs exert differential roles in different stem cells remain unclarified. However, current studies are insufficient as most results only reflected the transcript level of GAGs biosynthetic enzymes during different differentiation process. Glycomic analysis of stem cells in different lineage specification will provide the foundation for understanding the distinct roles of GAGs in different stem cells under specific physiological context.

### Mesenchymal Stem Cells

MSCs are one of the most popular adult stem cells with advantages including noninvasive sampling, sufficient supply, and fewer ethical concerns (Grayson et al., 2015; Abdel Meguid et al., 2018). Thus, MSCs have been widely applied in the treatment of different diseases and represents an attractive cell source for bone regeneration.

The expression of proteoglycans exhibits distinct spatiotemporal patterns during osteogenic differentiation. Upon osteogenic differentiation, HSPGs including syndecans (syndecan-1,-2,-3, and -4), glypican-1, glypican-3, and glypican-4 showed increased transcript levels, whereas only minimal increases were observed in chondroitin sulfate proteoglycans (CSPGs)/dermatan sulfate proteoglycans (DSPGs) including decorin (DCN), biglycan (BGN) as 2-O-sulfation and 6-O-sulfation of HS under osteogenesis as expression of N-sulfotransferase1/2 (NDST1/2), heparan sulfate 2-O-sulfotransferase (HS2OST) and heparan sulfate 6-O-sulfotransferase (HS6OST) increased under osteogenic induction (Haupt et al., 2009, 3). Zhao et al. also showed marked upregulation of heparan sulfate 6-O-sulfotransferase-3 (HS6ST3), which encoded enzymes that performed sulfation at the 6-O position in glucosamine in HS (Zhao et al., 2015). These data displayed a distinct profile of proteoglycans/GAGs profile in stem cells. Most analyses have been limited to transcriptomic analysis in proteoglycans biosynthesis, but these data may not reflect the true activity of core proteins as well as the exact composition of GAGs. Further study utilizing large-scale proteomic methods and advanced glycomic tools should be undertaken to provide better information to better understand distinct proteoglycans profiles as well as the regulation of proteoglycans synthesis during stem cell development (Li et al., 2016; Wu et al., 2019).

### Other Stem Cells

The dynamic profile of proteoglycans along with their sulfation patterns are also observed in other stem cells. Expression of several core proteins and their sulfotransferases exhibited significant changes in neural stem cells (NSCs) and differentiated neural cells. Upregulation of several HSPGs including *syndecan-4*, *glypican-1*, CSPGs including *aggrecan* and *decorin* was observed in the neuronal differentiation. *Glypican-4* and *glypican-6* mRNA were upregulated in astrocyte differentiation (Oikari et al., 2016). Apart from the expression profile of core proteins, biosynthetic enzymes of GAGs also indicates that sulfation patterns are different between undifferentiated and differentiated NSCs. In the differentiated neural cells, 2-O-sulfation increased as demonstrated by the higher expressions of *uronyl 2-O-sulfotransferase* and *C5-epimerase* mRNAs than NSCs. In contrast, 4-O-sulfation and 6-O-sulfation were lower than NSCs suggested by the decreased transcript level of *chondroitin 4-O-sulfotransferase*, *chondroitin 6-O-sulfotransferase*, and *N-acetylgalactosamine 4-sulfate 6-O-sulfotransferase* (Yamauchi et al., 2011). *In situ* hybridization also confirmed the expression of these enzymes in cells residing in adult neural stem cells niche. In order to provide a more direct and detailed profile of GAGs sulfation, (Akita et al., 2008) performed disaccharide analysis of neurospheres and E13 mouse brain cells and demonstrated that the synthesized CS/DS chains contained significant percentage of disaccharide units with 4-O-sulfation (Over 50%) and 6-O-sulfation. Functionally, degradation of CS by enzymatic treatment with ChABC led to reduced differentiation of radial glia to neurons as well as self-renewing radial glia (Sirko et al., 2007). Inhibition of GAGs sulfation by sodium chlorate also resulted in decreased number and size of neurospheres and disrupted cell cycle progression of NSCs (Akita et al., 2008; Schaberg et al., 2021). These results provide direct evidence that GAGs sulfation are essential regulator of NSCs homeostasis. Moreover, simple addition of specific GAGs chains (CS and Hep) failed to restore the normal size and number of neurospheres. These findings highlight that endogenous sulfation orchestrated by numerous enzymes allows for adaptive modification and plays an irreplaceable role in NSCs homeostasis.

Hematopoietic stem cells are capable of producing all blood cell lineages, which is essential for tissue regeneration (Pouzolles et al., 2016). Proteoglycans are key regulators of hematopoietic stem cell niche and modulate hematopoietic progenitor cell functions including adhesion, survival by binding and localizing growth factors to specific niches within the hematopoietic microenvironment (Siczkowski et al., 1992; Bruno et al., 1995). Several studies have confirmed that highly sulfated HS especially the N-sulfate rich domains are essential for hematopoiesis (Gallagher et al., 1983; Turnbull and Gallagher, 1988).A recent research demonstrated that exogenous 6-O-sulfate-rich bone marrow stromal cell-derived HS variant is capable of maintaining a subset of primitive HSCs during *ex vivo* expansion with improved clonogenicity and an increased potential to form erythroid and granulocyte progenitors (Bramono et al., 2011). Although current results indicates that GAGs are important in shaping the hematopoietic microenvironment, studies remain to be performed to better understand the fine regulation of GAGs structures including the developmentally orchestrated regulation of the biosynthetic enzymes and chains modifications during the homeostasis of hematopoietic stem cells. Furthermore, the results from different stem cells show that sulfated GAGs have distinct cell-specific roles in mediating the homeostasis of stem cells. For example, adequate sulfation level is required for NSCs to maintain self-renewal. Conversely, ESCs synthesize relatively low-sulfated GAGs and required sulfated GAGs to exit from self-renewal and commit lineage specification. Deciphering the cell-specific mechanisms of GAGs will provide more insight in the commitment of stem cell fate.

## Proteoglycans Modulate Stem Cell Self-Renewal

### GAGs Sulfation Influences Stem Cell Self-Renewal

Sulfation is a dynamic posttranslational modification; GAG sulfation patterns specifically affect the stemness of different stem cells. ESCs niches are distinguished by the presence of low-sulfated GAGs, whereas the sulfation level remarkedly increases as they undergo differentiation. Low-sulfation HS is mainly located on pluripotent cells, whereas highly sulfated HS is associated with differentiated cells (Kraushaar et al., 2013). *Ext-1* ^*−/−*^ ESCs (Kraushaar et al., 2010, 2012) and *Ndst1/2* ^*−/−*^ ESCs (Forsberg et al., 2012) were able to maintain in an undifferentiated state after long-term culture. There are also conflicting results observed in an *Ext1* knockdown ESCs cell lines prepared by RNA interference (RNAi), whereby HS deficiency showed suppressed potential for self-renewal and proliferation (Sasaki et al., 2008). These contradictory findings may be attributed to differences in the *Ext1* knockdown efficiency between these methods because there was still a small amount of residual HS-positive ESCs detected in the *Ext1* knockdown group. Also, the undersulfation of DS resulted in reduced activity of self-renewal marker alkaline phosphatase of D4ST1 KD mESCs, suggesting that sulfation contributes to the undifferentiated state of mESCs (Ogura and Nishihara, 2021). The currently available research does not provide detailed analysis of GAGs profiles including changes in GAGs composition and content. Thus, the potential requirement of a threshold HS/CS ratio or a proper ratio of HS/CS in maintenance of ESCs self-renewal cannot be ruled out.

ESCs are arrested in a naïve state and fail to exit from self-renewal in the presence of GAGs deficiency, while sulfated GAGs can facilitate GAG-deficient ESCs to exit from self-renewal. Exogenous treatment with HS or Hep were effective in rescuing the differentiation potential of ESCs along the neural and hematopoietic lineages. Sulfation is essential for restoring ESCs differentiation potential since no significant effect was detected compared to the control group (*Ext1* ^*−/−*^ ESCs with no treatment) when desulfated HS or Hep was added. These results support the hypothesis that the pro-differentiation ability of HS and Hep are sulfation-dependent (Holley et al., 2011; Pickford et al., 2011).

Specific sulfation patterns of GAGs are vital for promoting different lineage commitment of ESCs. For example, N-sulfation is vital for the neural specification of ESCs. Hep and HS can partially restore the percentage of Sox1^+^ NPCs by nearly 60%. Conversely, N-desulfated Hep was able to inhibit Sox1 acquisition by more than 20%, indicating the importance of the N-sulfate groups of Hep in promoting neural differentiation. Pickford et al., 2011). Similarly, N-sulfation and 6-O-sulfation of Hep were critical for hematopoietic differentiation of ESCs (Holley et al., 2011). Therefore, sulfated GAGs and their specific sulfation patterns are essential to allow ESCs to exit from self-renewal and commit to lineage specification.

Compared with ESCs, MSCs are multipotent and have a limited capacity for self-renewal. These fundamental differences indicate that they are mediated by a distinct regulatory network (Kléber and Sommer, 2004; Varga and Wrana, 2005; Semba et al., 2020). Therefore, the roles of GAGs and their sulfation in MSCs may differ from that of ESCs. Likewise, sulfation also affects the stemness of MSCs. HA, with no sulfate groups, was abundantly present in the MSCs niche (Qu et al., 2014) and prolonged the longevity of mouse MSCs (Chen et al., 2007). hMSCs cultured on HA-coated surfaces maintained high expression level of stemness markers (CD105, CD90) after a prolonged culture, and could preserve the differentiation potential up to 19 passages (Wong et al., 2017). However, unlike ESCs, highly sulfated GAGs were not inhibitors of MSCs self-renewal. For example, HS8, a HS variant with high affinity toward FGF-2, was decorated with 6-O-SO_3_ and bound and stabilized endogenous FGF2 to promote the proliferation of hMSCs through the FGFR1 signaling pathway. *In vitro* supplementation of HS8 upregulated the expression of genes preventing cells from aging (*CD74, CCL2, FANCD2, MDM2, SPRY2*) and downregulated the expression of genes inhibiting cell proliferation (*SULF2, CDKN2B*), maintaining the stemness and potency of hMSCs (Ling et al., 2020).

Briefly, GAGs and their sulfation patterns are critical for the homeostasis of both ESCs and MSCs. However, as the intrinsic nature of ESCs and MSCs differs, the underlying molecular mechanisms governing these two types of stem cells are distinct and cell-specific (Bhaskar et al., 2014; Huang et al., 2015). For example, FGF-2 functions as an inducer of differentiation in ESCs. Elevated sulfation of GAGs enabled better formation of the HS/FGF/FGFR complex and downstream signaling transduction, which ultimately led to differentiation of ESCs. Conversely, FGF-2 serves as potent stimulator for proliferation in MSCs. Thus, enhanced binding of HS with FGF-2 promoted proliferation in MSCs (Wijesinghe et al., 2017). Also, whether there are differential roles for endogenous and exogenous GAGs in different stem cell types remains unclear. In ESCs, studies have used gene knockout to evaluate the role of endogenously expressed GAGs. The phenotypes of these GAGs-deficient ESCs can be rescued by exogenous treatment of GAGs, indicating the possibility that endogenous and exogenous GAGs exert similar roles in ESCs homeostasis. In MSCs, only a few studies have constructed GAGs-deficient models using GAGs enzymatic depletion, whereas the role of exogenous GAGs is widely studied as a coating materials or culture adjuvant which is usually modified with a specific sulfation pattern (Manton et al., 2007; Wijesinghe et al., 2017). Therefore, the currently available studies are insufficient to determine whether exogenous and endogenous GAGs exert a differential effect on different stem cells types as well as whether they act on stem cells via different mechanisms.

### GAGs-Regulated Signaling Transduction in Stem Cell Self-Renewal

Accumulating evidence has suggested that GAGs regulate stem cell homeostasis by continuously adapting their structures to selectively bind to signaling molecules (Table 1). The intricate structure of GAGs endows them with this essential biofunction. *Ext-1* ^*−/−*^ ESCs were not capable of exiting from self-renewal due to HS deficiency (Kraushaar et al., 2010), which resulted in hypoactivation of FGF-FGFR signaling. This was further supported by a successful replication of the *Ext-1* ^*−/−*^ ESCs phenotype in an FGF-inhibited cell model. The inhibition of FGF induced phosphorylation of ERK1/2 was also observed when the HS antagonist surfen (Huang M. L. et al., 2018) or the sulphate inhibitor NaClO_3-_ was applied (Lanner et al., 2010). Conversely, supplementing exogenous HS can stimulate stem cells to produce more FGF-2. Exogenous HS-8, which is characterized by high affinity with FGF-2, can bind to FGF-2 quickly by competing with endogenous HS, enabling FGF-2 to spread to other cells and exert its biological activity (Titmarsh et al., 2017).

**TABLE 1:** Phenotypes of proteoglycan-deficient stem cells.

Cell type	Proteoglycans affected	Phenotype	Mechanism	(References)
*Ext-1* ^*−/−*^ ESCs	HSPGs	When cultured with no or low concentration of leukaemia inhibitory factor (LIF), *Ext-1* ^*−/−*^ ESCs maintained the typical morphology of ESCs, high ALP activity and high expression of the pluripotency gene *Nanog* and were unable to exit from self-renewal	FGF and BMP signaling	(Kraushaar et al., 2010, 2012)
*GlcAT-I* ^*−/−*^ ESCs	HSPGs, CSPGs, DSPGs	*GlcAT-I* ^*−/−*^ ESCs failed to initiate differentiation and showed higher expression of two pluripotency genes *Nanog* and *Sox2* than *GlcAT-I* ^*+/-*^ ESCs and *GlcAT-I* ^*+/+*^ ESCs	CS colocalizes with and binds to E-cadherin	Izumikawa et al. (2014)
*Ndst1/2* ^*−/−*^ESCs	Sulfated proteoglycans	*Ndst1/2* ^*−/−*^ESCs can take the initial step toward differentiation into all three germ layers but were arrested in a primitive ectoderm and/or endoderm stage	FGF signaling	Lanner et al. (2010); Forsberg et al. (2012)
		*Ndst1/2* ^*−/−*^ESCs blocked differentiation and were maintained in a naïve state	FGF4 signaling	
*Ext-1* knockdown cancer stem cells	HSPGs	Knockdown of *Ext-1* in MCF7/ADR cells significantly reduced cancer stem cell markers, mammosphere number and the colony formation ability	FGF4 signaling	Manandhar et al. (2017)
*Ext-1* ^*−/−*^ prostate stem/progenitor cells (PrSCs)	HSPGs	Deletion of *Ext-1* in PrSCs disrupted their ability to self-renew and attenuated prostate regeneration	TGF-β signaling	Rai et al. (2020)
Surfen treated ESCs	HSPGs	Surfen treated ESCs were arrested in their pluripotent state due to decreased binding sites for growth factors within their GAG chains	FGF2/MAPK, RTK, and VEGF signaling	Huang et al. (2018b)

HSPGs, heparan sulfate proteoglycans; CSPGs, chondroitin sulfate proteoglycans; DSPGs, dermatan sulfate proteoglycans; FGF, fibroblast growth factor; BMP, bone morphogenetic protein; FGF4, fibroblast growth factor 4; FGF2, fibroblast growth factor 2; TGF-β, transforming growth factor-β; MAPK, mitogen-activated protein kinase; RTK, receptor tyrosine kinase; VEGF, vascular endothelial growth factor.

As two major categories of HS proteoglycans, sydencans and glypicans participate in the regulation of FGF2 signaling by stabilizing the molecular assembly of growth factors with their receptors (Smock and Meijers, 2018). Specific sulfation groups of HS such as NS and 2S residues lay the foundation for the assembly of HS with FGF2, while the 6S residue is the key to facilitating the bond between FGF2 with FGFR1 and subsequent activation of downstream intracellular signaling (Yamada et al., 2017; Qiu et al., 2018). N-sulfation of GAGs plays a vital role in the regulation of fibroblast growth factor 4 (FGF4) signaling. *Ndst-1/2* ^*−/−*^ ESCs were unable to bind to FGF4, resulting in decreased ERK1/2 phosphorylation and self-renewal dysfunction due to the lack of N-SO_3_ (Lanner et al., 2010). In addition to the FGF family, it has been proposed that GAG chains participate in the regulation of Wnt signaling via a restricted diffusion mechanism, in which Wnt ligands are bound by HS chains and transported by repeated association and dissociation (Wang et al., 2019). Endosulfatase selectively removes the 6-O-sulfate groups from HS proteoglycans and release Wnts for binding with Frizzled and the low density lipoprotein receptor related proteins 5/6 (LRP5/6) receptor, thereby activating the subsequent signaling. The accumulation of β-catenin inside the cell nucleus maintains the stemness and potency of MSCs (Dhoot et al., 2001; Morimoto-Tomita et al., 2002; Ai et al., 2003).

As such, substantial studies have revealed that proteoglycans and GAGs modulate stem cell behavior through their interaction with growth factors and their corresponding receptors in multiple signaling pathways. Exogenous addition of GAGs of different sulfation patterns showed differential effects in restoring the lineage specification potential of *Ext1* ^*−/−*^ESCs. The exogenous addition of modified Hep and HS indicated that the N-sulfation is vital for promoting the percentage of Sox1^+^ NPCs (Pickford et al., 2011). Moreover, *Ext1* ^*−/−*^ESCs were unable to differentiate into hematopoietic lineages partially due to an impaired response to BMP4. Using a range of chemically modified Heps, Holley et al. (2011) determined that N-sulfation and 6-O-sulfation of Hep was critical for rescuing the potential for hematopoietic differentiation of *Ext1* ^*−/−*^ESCs. Also, Hep addition restored the activity of multiple signaling pathways including BMP with activation of pSMADs, highlighting a critical role for HS as a co-receptor in the BMP4 signaling pathway. These results indicated that modifications of GAGs are essential for stem cell differentiation since they are critical for interacting with growth factors that trigger or activate the differentiation process. However, due to the structural heterogeneity of GAGs, greater efforts should be undertaken to better demonstrate the role of different types of GAGs in facilitating or inhibiting a specific signaling pathway. Moreover, the roles of sulfate groups as well as endosulfatases in shaping GAGs sulfation and their subsequent influence on stem cell pluripotency also awaits further exploration.

## Proteoglycans Modulate the Osteogenic Differentiation of Mesenchymal Stem Cells

Proteoglycans are closely related to the development and regeneration of bone tissue. A large number of studies have observed that DCN, BGN, perlecan (PLN), and aggrecan are widely and differentially expressed in different stages of bone tissue development (Kamiya et al., 2001; Domowicz et al., 2009; Ishijima et al., 2012). The complete structure and function affect complex bone tissue development and its regulation network. The regulatory role of core proteins has been elucidated from proteoglycan-deficient animal models that exhibited reduced skeletal growth and bone mass, in addition to abnormalities in collagen fibrils (Table 2). Moreover, core proteins widely participate in osteogenic differentiation of MSCs by mediating multiple signaling pathways including transforming growth factor-β (TGF-β), bone morphogenetic protein (BMP) and Wnt pathways (Table 3.). Furthermore, GAGs are also highly involved in osteogenesis., Liu et al*.* (2018) observed abnormal development of craniofacial bone characterized by deficient bone mineralization and significantly enlarged cranial sutures by constructing a GAGs-deficient mouse model, in which they specifically knocked out *FAM20B* gene in neural crest derived-MSCs. The exogenous addition of GAGs enhanced the osteogenic differentiation of MSCs, resulting in upregulation of osteogenic markers (Uygun et al., 2009) and accelerated bone healing processes (Förster et al., 2017). These studies suggested that the multiple functions of proteoglycans are related to both its core protein and GAGs. However, the roles of GAGs are far more complex.

**TABLE 2:** Proteoglycans deficient animal models with skeletal phenotypes.

Proteoglycan	Phenotype in hard tissues	(Refs.)
Decorin (DCN)	No significant change in the skeletal system was found in *Dcn* ^*−/−*^ mice Corsi et al. (2002) nor mutant mice with no DS chains attached to *Dcn* Moffatt et al. (2017). However, abnormalities of collagens fibril in bone were observed including decreased diameter and size of collagens fibrils	(Corsi et al., 2002; Moffatt et al., 2017)
Biglycan (BGN)	Disruption of *biglycan* gene resulted in reduced skeletal growth and bone mass leading to osteopenia Corsi et al. (2002)	Corsi et al. (2002)
Decorin, Biglycan	*Dcn*/*Bgn* double-knockout mice exhibited a more striking and early appearing skeletal phenotype including shorter and wider long bones and marked osteopenia which was barely detectable in single mutant animals but was markedly detectable in double-knockout mice at 2 months of age. Reduced overall collagen mass was observed in bone Corsi et al. (2002)	Corsi et al. (2002)
Fibromodulin	The lack of fibromodulin impaired dentin mineralization, increased the diameter of collagen fibrils in the predentin and delayed enamel formation	Goldberg et al. (2006)
Glypican-3 (GPC-3)	GPC3-knockout mandibles were larger than wild-type mandibles for all dimensions	Mian et al. (2017)

**TABLE 3:** Proteoglycans are involved in osteogenic differentiation.

Proteoglycan	Role in osteogenic differentiation	(Refs.)
Glypican-3	Increased expression of the GPC-3 core protein was observed during the osteogenic differentiation of MC3T3-E1 cells. *Gpc3* knockdown abrogated the expression of Runx2 and thereby suppressed osteogenic differentiation of MC3T3-E1 cells	(Haupt et al., 2009, 3)
Perlecan (PLN)	Exogenous addition of PLN promoted osteogenic differentiation of MSCs whereas blocking of intrinsic PLN resulted in reduced calcium apposition	Nakamura et al. (2014)
Biglycan	Overexpression of BGN promoted the osteogenic differentiation of MSCs as evidenced by increased ALP activity and upregulated expression of osteoblast specific marker genes such as *Runx2*, *OCN,* and *Col1* through activation of TGF-β signaling pathway Wu et al. (2013)	Chen et al. (2004); Wang et al. (2010); Wu et al. (2013)
	BGN promoted osteoblast differentiation through ERK activated Runx2 pathway, and through the Smad signaling pathway. Overexpression of BGN in MC3T3-E1 cells also promoted mineralization Wang et al. (2010)	
	BGN has also been reported to promote bone morphogenetic protein-4 (BMP-4) stimulated osteoblastic differentiation via its GAGs chains. BGN deficiency caused less BMP-4 binding and reduced core-binding factor α1 (Cbfa1) expression and ultimately affected osteoblast differentiation (Chen et al. (2004), 4)	
Decorin	Overexpression of *Dcn* in MC3T3-E1 resulted in delay of mineralization and thinner collagen fibril whereas silencing *Dcn* led to accelerated mineralization and a greater number of mineralized nodules, and thicker collagen fibril with larger diameters and irregular direction	Mochida et al. (2009)
Keratocan (KERA)	*Kera* ^*−/−*^ primary calvarial cell showed reduced expression of mature osteoblast differentiation markers, such as BSP and OCN. And *Kera* ^*−/−*^ mice had a significantly decreased rates of bone formation and mineral apposition	Igwe et al. (2011)
Proline/arginine-rich end leucine-rich protein (PRELP)	The expression of PRELP increased with the osteogenesis induction of preosteoblastic MC3T3-E1 cells. Down-regulation of PRELP expression by shRNA reduced ALP activity, mineralization, and expression of osteogenic marker gene *Runx2* and suppressed osteogenic differentiation	Li et al. (2016)
Osteoadherin (OSAD)	OSAD was upregulated during osteogenic differentiation of hMSCs (Im et al., 2016). Overexpression of OSAD resulted in an increase of osteoblast differentiation features, such as increased ALP activity and increased *in vitro* mineralization. This suggested that OSAD overexpression enhanced the differentiation and maturation of osteoblasts	Rehn et al. (2008)
Osteoglycin (OGN)	Overexpression of OGN promoted osteogenic differentiation as evidenced by the increased levels of Wnt5b, Runx2, OCN, ALP and Col1 as well as bone formation Chen et al. (2017), suggesting that OGN might positively promote osteogenic differentiation	Im et al. (2016)
	*OGN* stably overexpressed in MC3T3-E1 cells showed significantly decreased level of Runx2 and Osterix expression, indicating that *OGN* might serve as suppressor for early differentiation of osteoblastic progenitors Im et al. (2016)	Chen et al. (2017)
Betaglycan	The disruption of betaglycan in MSCs completely blocked osteogenic differentiation via elevated Wnt signaling	Cook et al. (2019)

Runx2, runt-related transcription factor 2; OCN, osteocalcin; COL1, collagen type 1; ALP, alkaline phosphatase; BSP, bone sialoprotein; TGF-β, transforming growth factor-β.

To determine the contribution of GAGs to the functional properties of proteoglycans *in vivo*, Moffatt et al. (2017) generated a mutant mouse whose DCN lacked a DS chain by substituting an alanine for serine at the DS attachment site of DCN. Surprisingly, the body size and limb length were similar between groups. All connective tissues appeared to be normal upon histological examination and no abnormalities were found in the structure of collagen fibrils (Moffatt et al., 2017). The findings suggested that there was a compensatory mechanism between proteoglycans, which explained the discrepancies in phenotypes between mutant mice lacking the DS chains on DCN and GAGs-deficient mice. This compensatory mechanism has been mostly studied between the two SLRPs members DCN and BGN. *Bgn*/*Dcn* double-deficient mice have a more severe phenotype in both the long bone and skin compared to wildtype or singly deficient SLRP mice. *Bgn*/*Dcn* double-deficient mice exhibit a more striking and early appearing skeletal phenotype including shorter and wider long bones and markedly osteopenia which is barely detectable in single mutant animals (Corsi et al., 2002). Further analysis also revealed a similar compensatory up-regulation of *BGN* gene expression in *Dcn*-deficient mice but *DCN* up-regulation was not observed in *Bgn*-deficient mice (Zhang et al., 2009b). Immunohistochemistry revealed that in the absence of BGN, DCN is up regulated throughout the PFS (Wadhwa et al., 2007). However, *in vitro* analysis did not reveal differences in transcriptional levels of *BGN* in MC3T3-E1 derived clones expressing either higher or lower levels of *DCN* (Mochida et al., 2009). Similarly, in cell clones expressing higher and lower levels of *BGN*, only slight changes of *DCN* levels were detected in the culture media (Parisuthiman et al., 2005).

### GAGs Sulfation Affects the Osteogenic Differentiation of Stem Cells

As described above, the sulfation pattern of GAGs undergoes continuous changes during osteogenic differentiation. The sulfation level of GAGs is positively related to the differentiation of SCs as differentiating cells expressed highly sulfated GAGs (Nairn et al., 2007; Kraushaar et al., 2013). Highly sulfated GAGs are decorated by abundant sulfate groups such as N-SO_3_, 2-O-SO_3_, and 6-O-SO_3_ on HS and 2-O-SO_3_, 4-O-SO_3_, and 6-O-SO_3_ on CS/DS. These sulfate groups provide binding sites for multiple growth factors (Smock and Meijers, 2018) and help shape morphogen gradients (Balasubramanian and Zhang, 2016).

HS is the most studied class of GAGs given its modulation of a wide range of biological processes. The structural and functional diversity of HS are conferred by the modification of sulfation at the C2, C6, and C3 positions of uronic acid and at the N position of glucosamine. These sulfate groups are the structural foundation for cellular interaction as well as the affinity sites for HS binding for a range of protein ligands (Köhling et al., 2019; Zhang et al., 2019). N-sulfation provides critical binding sites for bone morphogenetic protein 2 (BMP-2) and are necessary for subsequent downstream signaling. The loss of the N-sulfate group remarkedly diminished the sequestration of BMP-2 and resulted in nearly no calcium deposition *in vitro* and reduced amount of newly formed bone tissue *in vivo* due to decreased Smad 1/5/8 phosphorylation (Smith et al., 2018). N-sulfation was also essential for Wnt3a binding as well as the formation of Wnt3a-HEP complexes, and enhanced osteogenesis via PI3K/Akt/RUNX2 pathway. Depletion of N-sulfation markedly reduced alkaline phosphatase (ALP) activity which was induced by Wnt3a-HEP complexes (Ling et al., 2010). Apart from facilitating the interaction between ligands and their corresponding receptors, HS also reduced interactions with the BMP-2 antagonist Noggin (Murali et al., 2013) and the Wnt inhibitors dickkopf 1 (DKK1) and sclerostin (SOST) (Simann et al., 2015). However, the role of N-sulfation in reducing signaling pathway inhibitors is unknown.

The 6-O-sulfation of HS chains was also found to be positively associated with to the osteogenic differentiation of MSCs (Zhao et al., 2015). Knockdown of *HS6ST3* in MSCs remarkedly impaired osteogenic differentiation, thus halving ALP activity, and reduced the expression of osteogenic markers such as *OCN* and *RUNX2* by 60 and 75%, respectively. Although the underlying mechanisms remain unclear, evidence has shown that 6-O-sulfation is relevant to the regulation of several signaling pathways. For instance, 6-O-sulfation promotes FGF2/ERK signaling (Chanalaris et al., 2019), and inhibits Wnt (Gao et al., 2016) signaling in some cellular processes but studies investigating its role in signaling transduction during osteogenic differentiation are limited.

Apart from 6-O-sulfation, 2-O-sulfation has also been implicated in osteogenesis. Upon secreting growth factors that are essential for differentiation, MC3T3-E1 cells simultaneously produce sulfated GAGs to stabilize growth factors and enhance their biological functions. The GAGs derived from differentiating MC3T3-E1 cells present significantly higher affinity for BMP-2 and basic fibroblast growth factor (bFGF) (Fukunishi and Tabata, 2018). The enhanced affinity for growth factors could be attributed to increased content of 2-O-sulfation validated by disaccharide analysis of secreted GAGs (Fukunishi and Tabata, 2018). These findings indicated that GAGs enriched with 2-O-sulfation were upregulated to facilitate osteogenic differentiation by binding with growth factors.

Consistent with the forementioned results, highly sulfated CS promoted osteogenic differentiation. Oversulfated CS-E which possesses 4,6-disulfates in N-acetyl-galactosamine, was found to be significantly upregulated in the bone matrix during osteogenic differentiation, and the enzymatic digestion of CS resulted in impaired formation of mineral modules (Miyazaki et al., 2008). Further, CS-E is a ligand for bone morphogenetic protein 4 (BMP4) (Miyazaki et al., 2008), N-cadherin, and cadherin-11 (Koike et al., 2012). By binding to N-cadherin and cadherin-11, CS-E decreased ERK1/2 phosphorylation, activated Smad3 and Smad1/5/8 leading to enhanced osteogenesis (Koike et al., 2012). Notably, monosulfated CS-A, CS-B with 4-sulfation, and CS-C with 6-sulfation did not enhance binding to BMP-4 (Miyazaki et al., 2008) nor did N-cadherin or cadherin-11 (Koike et al., 2012). Altogether this suggested that 4,6-disulfates in N-acetyl-galactosamine provided binding sites for these protein ligands. Collectively, these data suggest that a certain degree of sulfation is necessary for signaling transduction during osteogenic differentiation.

## Proteoglycans Modulate the Chondrogenic Differentiation of Mesenchymal Stem Cells

Proteoglycans are a major component of cartilage and are extensively involved in cartilage development and regeneration. Extensive studies have demonstrated that DCN, syndecan, PLN, and aggrecan were widely and differentially expressed in different stages of chondrogenesis (French et al., 1999; Prante et al., 2006). In addition, proteoglycans are required for the modulation of chondrogenic process by mediating signaling transduction including BMP, TGF-β, and Wnt signaling pathways (Fisher et al., 2006; Chen et al., 2016; Wang et al., 2019). Syndecan-3 modulates the interaction of BMP2 and its receptors thereby limiting the strength of BMP signaling during limb cartilage differentiation (Fisher et al., 2006). Further, PLN deficiency led to reduced cartilage matrix production and Sox9 and Col2a1 mRNA levels *in vitro* and impaired the incorporation of newly synthesized ECM *in vivo* (Sadatsuki et al., 2017; Ar et al., 2020). Apart from core proteins of proteoglycans, GAGs are also highly involved in chondrogenesis. CS content significantly increased during chondrogenic differentiation from about 60% to nearly 90% at day 21 whereas the percentage of HS and HA decreased (Silva et al., 2020). Furthermore, through conditionally ablating the Ext1 in limb bud mesenchyme, Matsumoto et al. found that Ext1 mutant mice displayed severe limb skeletal defects including shortened and malformed limb bones, and the chondrogenic differentiation of *Ext1* ^*−/−*^ MSCs was delayed and impaired (Matsumoto et al., 2010). Furthermore, knockout of *galnact1* (t1) and *Csgalnact2* (t2) in cartilage revealed disrupted endochondral ossification and impaired chondrocyte proliferation (Shimbo et al., 2017).

The biological roles of GAGs in chondrogenesis may also be closely related to their sulfate patterns and levels. Undersulfation of CS in the limb growth plate led to diminished Indian hedgehog (Ihh) signaling and abnormal Ihh protein distribution in the ECM (Cortes et al., 2009). Mutation in the sulfotransferase genes led to cartilage and bone abnormalities, highlighting the significance of sulfation patterns of GAGs in normal skeletal development (Klüppel et al., 2005). Sulfotransferase are responsible for transferring sulfate to the CS backbone and for synthesizing CS with different degrees of sulfation and specific sulfation patterns. Mutation in the gene encoding C6ST-1 leads to significantly reduced 6S and 2-6S together with marked undersulfation of CS in spondyloepiphyseal dysplasia (SED) Omani type patients (Thiele et al., 2004). Moreover, ablation of the C4ST1 gene resulted in severe chondrodysplasia characterized by disorganized cartilage growth plate. A more detailed analysis revealed an abnormal disruption in BMP levels and strong activation of TGF- signaling in the developing skeleton and cartilage growth plate, suggesting that 4S is indispensable for modulation of balanced signaling transduction and cartilage growth plate morphogenesis (Klüppel et al., 2005). Exogenous addition or scaffold materials using specific GAGs also indicated that sulfation pattern of GAGs in promoting chondrogenesis, where CS-C and CS-E were more capable of enhancing chondrogenic differentiation (Kawamura et al., 2014; Menezes and Arinzeh, 2020). These findings confirm that both sulfation level and sulfation patterns play a critical role in chondrogenesis.

### GAGs-Based Materials in Bone Tissue Regeneration

As natural components of the ECM, GAGs possess many advantageous characteristics including biocompatibility, degradability, and non-immunogenicity, making them attractive candidates for biomedical applications for drug delivery and tissue engineering. GAGs are essential components of stem cell microenvironments, providing structural supports for cells and docking sites for various signaling molecules. This paradigm also applies to osteogenesis, as bone cells constantly interact with their microenvironment during osteogenic differentiation, ECM deposition and biomineralization. GAG-based materials have been developed in a variety of forms including hydrogels, nano-and micro-particles, surface coatings, and scaffolds. In addition, the modification of GAGs greatly enhances their biological functions including anti-inflammatory and pro-osteogenesis potentials (Hempel et al., 2014a).

### Unsulfated GAGs

#### Pro-osteogenesis Potential

HA is the only GAGs with a linear and unbranched structure without any sulphate groups. Its high molecular mass and large hydrodynamic volumes influence the biomechanical properties of the ECM, tissue hydration, receptor clustering, and receptor-ligand interactions. On the cell surface, HA interacts with a wide range of HA-binding proteins to influence cell proliferation and differentiation, migration, angiogenesis, and inflammation.

Although many studies have shown that HA holds great potential for promoting osteogenesis *in vitro* (Koca et al., 2019; Yuan et al., 2019; Jang et al., 2020), recent findings have demonstrated that HA serves more as an osteoinductive scaffold since application of HA alone failed to induce sufficient bone regeneration as compared to treatments involving graft materials (Sindel et al., 2017; Diker et al., 2018). Similarly, the presence of HA alone in the implant osteotomy also failed to yield improved osseointegration (Yazan et al., 2019). Arpağ et al*.* found that when combined with xenografts, HA contributed to new bone formation but did not improve the quality of newly formed bone (Arpağ et al., 2018). This suggested that HA alone was insufficient for bone regeneration, therefore the combination of HA and other material such as collagen and hydroxyapatite may be essential for improving bone regeneration *in vivo*. Yuan et al. (2019) developed a bio-degradable bone graft material consisting of multiarm polyethylene glycol crosslinked with HA hydrogels, which brought a significant improvement to ALP activity and calcium mineralization *in vitro*. The multiarm PEG-HA hydrogels also facilitated healing of the cranial bone defects of rats. Modification of HA is also utilized to facilitate bone repair. The HA and collagen (Col) are two of the major components of the ECM, showing potential to be used as a template for biomineralization. Li M. et al. (2019) developed a biomimetic nanofiber network based on Col/oHAs and its mineralized product. The results indicated that the oHAs-based scaffolds promote the attachment of endothelial cells and facilitate the osteogenic differentiation of MC3T3-E1 and BMSCs.

#### Pro-chondrogenesis Potential

As the major GAGs found in cartilage, HA are extensively involved in a variety of biological processes such as increasing the chondrogenic differentiation potential of MSCs as well as supporting ECM production (Dvorakova et al., 2008; Sato et al., 2014; Alessio et al., 2021). Moreover, HA shows great promise as a chondrogenic adjuvant in stem cell-based therapy for cartilage repair (Wong et al., 2021). Compared to other hydrogels such as collagen hydrogel, HA hydrogels facilitate chondrogenesis by creating a relatively stable physical microenvironment for MSCs and supported continuous production of cartilage-related matrix (Yang et al., 2021).

HA is also easily modified or crosslinked with other biomolecules and including peptides and RGD sequences for promoting proliferation, adhesion, and greater chondrogenic differentiation (Cavalli et al., 2019; Teng et al., 2021). Modifications of HA hydrogel by aldehyde groups and methacrylate (AHAMA) on the polysaccharide backbone significantly improve its durability and stability under humid environments as in native cartilage. AHAMA hydrogel exhibit enhanced proliferation and migration of BMSCs *in vitro*. Furthermore, through the incorporation of aldehyde groups and methacrylate AHAMA hydrogel can localized on the cartilage surface with multiple anchoring mechanisms, which significantly promote integration between neo-cartilage and host tissues, and significantly improved cartilage regeneration *in vivo* (Chen et al., 2021a). Similarly, HA cross-linked with the transglutaminase (TG) can attach covalently to fibrinogen and fibrin, ensuring good tissue adhesion (Broguiere et al., 2016), which is essential for long-term success for cartilage regeneration *in vivo*. In an ovine osteochondral defects model, HA-TG adhered to the native tissue and facilitated the recruitment and infiltration of MSCs. It also preserved the adjacent cartilage, providing a favorable environment for the generation of a neocartilage tissue (Levinson et al., 2021).

Apart from chondrogenic property, HA-based hydrogels have also been optimized as carriers for sustained drug delivery for chondrogenesis under inflammatory environments, which is desirable for the long-term treatment for osteoarthritic joints. Jin et al. (2016) combined epigallocatechin-3-gallate (EGCG) with tyramine-conjugated HA and gelatin to control inflammation and enhance cartilage regeneration. EGCG not only has an anti-inflammatory effect as it protected chondrocytes against the pro-inflammatory factor, IL-1β, but it also enhanced chondrogenic regeneration *in vitro* and *in vivo*. Furthermore, Ziadlou et al. (2021) utilized HA enzymatically crosslinked silk-fibroin/hyaluronic acid-tyramine composite hydrogels (HA-SF) to carry the anti-inflammatory drugs vanillic acid (VA) and epimedin C (EpiC) and achieved sustained release over 60 days.

From the above evidence, it appears that HA-based materials can be optimized through a wide range of modifications and these products have great potential and opportunities for promoting improvement of osteochondral defects and reducing inflammation.

#### HA as Delivery Agent for Anti-inflammation

HA is also an encouraging drug delivery agent for local orthopedic implants that are frequently the subject of complications caused by local aseptic inflammatory reactions and bacterial infections (Passi and Vigetti, 2019). Immobilized HA on the surface of titania nanotubes (Ti-NTs) greatly enhanced the biological activity of the implant and slow down the release rate of the drug in Ti-NTs. In one study, a multilayer coating consisting of Col and HA on the Ti-NT surface was loaded with the antibacterial drug enoxacin (EN). This Col/HA coating provided consistent and controllable drug release that lasted for more than 7 days, which significantly improved the release kinetics of drugs in the tubes compared with the control group (Ti-NT + EN) (Li H. et al., 2019). The high hydration capacity of HA has also been applied to biofilm repelling for its high surface energy. Combined with a load of antibacterial drug triclosan (TRI), the multilayer coating released about 25% of loaded TRI within the initial period of bacterial adhesion and continued as a bactericide reservoir, which made them potential biomaterials for both inhibiting bacterial adhesion and restricting bacteria viability during the critical post-implantation period (Valverde et al., 2019). As such, HA could promote osteogenic differentiation by providing an adaptable environment and optimized drug effects through controllable long-term release, making it an excellent candidate for bone tissue engineering approaches.

### Sulfated GAGs

Sulfated GAGs have been widely used in constructing bone tissue engineering scaffolds for their pro-osteogenesis and pro-chondrogenesis potential (Förster et al., 2017; Meghdadi et al., 2019; Singh et al., 2020). In addition, the incorporation of sulfated GAGs into hybrid scaffolds can fine-tune growth factor binding and achieve controlled release so as to continuously release cellular signals for promoting bone healing and regeneration (Anjum et al., 2016; Huang B. et al., 2018).

#### Pro-osteogenesis Potential

Various aspects of sulfated GAGs, including modifications and administration concentrations can be adapted to optimize their potential. The negatively charged sulfate groups on GAGs created an osteogenic suitable environment by binding positive calcium and phosphate, which is dependent on GAGs concentrations. To determine the optimized concentration of GAGs, Kim et al*.* (2017) constructed a PEGDA/CS-based hydrogel with various concentrations of CS (0–10%) and identified a positive correlation between CS levels and calcium phosphate binding. The authors found that 10% CS hydrogel induced relatively higher expression of *RUNX2* (9 times higher), *COL1* (6 times higher), and *ALP* (50 times higher) *in vitro*. Transplantation of the cell-laden hydrogels showed threefold higher regenerated volume and was integrated with native bone tissue. In contrast, although loading of HS also resulted in positive effects in bone repair, no differences were found between low and high concentration loading (50 μg/ml or 500 μg/ml) (Liu et al., 2020). *In vitro* experiments showed that low concentrations of HS exhibited the best effect on promoting cell adhesion and differentiation, whereas high concentrations of HS resulted in the inhibition of cell proliferation. The discrepancies might arise from the complex functions between GAGs as well as the differences between components, and mechanical and biochemical properties of these scaffolds.

Modifications are another effective approach to improve the biological functions of GAGs-based scaffolds, and include sulfation and oxidation. Oversulfation of GAGs has been validated for reducing inflammation and promoting new bone formation. When cultured on an artificial ECM made of collagen and oversulfated GAGs derivates, the inflammatory response of hMSCs was inhibited. The formation of pro-inflammatory mediators such as IL-6, IL-8, monocyte chemoattractant protein-1, and prostaglandin E2 was reduced. In addition, downstream events such as nuclear translocation of NFkB and expression of pro-inflammatory mediators and COX-2 were abrogated (Hempel et al., 2014a). Furthermore, the artificial ECM promoted the osteogenic differentiation of hMSC as indicated by an increased activity of tissue non-specific alkaline phosphatase (TNAP) and calcium deposition (Hempel et al., 2014a; 2014b). Selective desulfation of GAGs was proven suitable for creating adjustable signaling gradients and effectively controlling the fate and morphogenesis of MSCs *in vitro* (Atallah et al., 2018). Oxidization of GAGs (oGAGs) also improved the osteoinductive potential of scaffolds due to their higher thickness and roughness compared to the native counterparts and were capable of controlled release of BMP-2 (Anouz et al., 2018).

GAGs analogues, such as GAGs variants and GAGs mimics, are potential tools as they were designed to sequester and stabilize growth factors better and to overcome the drawbacks of native GAGs including their natural heterogeneity in structure, difficulty in modification, and uncertain biological roles (Tansik et al., 2016). Quang Le et al. (2021) synthesized an HS variant with high affinity toward BMP-2 named HS3 and constructed an HS3-functionalised scaffold integrated with collagen and bone granules with the aim to deliver BMP2 with sustained release. These scaffolds were able to retain up to 58% of the initial amount of BMP2 over 27 days, approximately 3-fold higher than scaffolds without HS3, while sustaining the bioactivity of the retained BMP2. By incorporating Hep-mimicking polysulfonates, poly-vinylsulfonic acid (PVSA) or poly-4-styrenesulfonic acid (PSS) into the MeGC hydrogel, Kim et al*.* (2018)observed a controllable release of ∼30% of loaded BMP-2 compared to ∼60% of the control group over a 21-day period, which resulted in a better osteogenic ability as indicated by elevated ALP activity and transcript levels of osteogenic markers.

#### Pro-chondrogenesis Potential

GAGs are major components of the cartilage matrix. CS contributes to 70–80% of the GAGs in cartilage with chondroitin-6-sulfate and chondroitin-4-sulfate accounting for 60% and 10–20%, respectively (Taylor and Miller, 2006). CS is now extensively studied and has attracted increasing attention as a potential biomaterial for cartilage tissue engineering. CS-based materials support chondrocyte matrix deposition and chondrogenic differentiation of MSCs as well as prevent further differentiation into hypertrophic chondrocytes (Varghese et al., 2008; Meghdadi et al., 2019; Alessio et al., 2021). As other types of GAGs including DS and KS are only composed of a small percentage of the cartilage matrix, studies regarding their biological potential as materials for cartilage tissue regeneration remain to be elucidated in the future.

CS is now widely functionalized into formats with adhesive properties which facilitate better preservation of adjacent cartilage tissue that are essential for recruitment of endogenous MSCs. Using mussel adhesive-inspired catechol chemistry, Shin et al. developed a functional CS hydrogel (CS-CA hydrogels) that exhibits significantly superior adhesive properties (∼3 N) over conventional CS hydrogels (and ∼0.05 N) thereby enhanced cartilage integration with host tissue and neo-cartilage formation. In addition, CS-CA hydrogels promote chondrogenic differentiation of MSCs by providing a cartilage-like microenvironment (Shin et al., 2021). Moreover, through modification or crosslink with other biomolecules, CS-based materials are not only designed for better recapitulation of the MSCs niche to promote chondrogenesis but can also preserve the immunosuppressive potential of MSCs and mediate repair under inflammatory conditions. By crosslinking CS onto a collagen-based scaffold (CSCL), this biomimetic scaffold was able to reduce inflammation *in vivo* by limiting lymphocytic infiltration (Corradetti et al., 2016). Moreover, crosslinking of CS into a CS-functionalized scaffold (CSS), CS efficiently suppressed the production of pro-inflammatory cytokines (NO and PGE2) and reduced the expression of their inducible enzymes PGES and iNOS (Chen et al., 2021b). Therefore, CS act as protective agent by decreasing expression of pro-inflammatory cytokines and plays an important role in moderating cartilage repair under inflammation.

CS are known to interact with numerous growth factors electrostatically via negatively charged sulfate groups. Regulating the sulfation level of GAGs is a promising approach for controlled growth factor delivery and release. Desulfation of CS significantly increased the release of histone by 1.5-fold over 8 days compared to natively sulfated CS, whereas sulfated CS was able to sequester greater amount of growth factor TGF-β1. Furthermore, chondrogenic differentiation was more marked in desulfated-CS hydrogels in the presence of TGF-β1 since sulfated CS binds TGF-β1 and decreased the effective concentration exposed to MSCs. These findings demonstrate that sulfation level can be utilized as powerful tool to modulate growth factor delivery and release as well as regulate MSCs response to soluble cues (Lim and Temenoff, 2013).

Collectively, GAGs are widely used in cartilage and bone tissue engineering for their multifactorial roles including promotion of osteogenesis, chondrogenesis, drug delivery and sustained released of growth factors. The biological functions of GAGs-based scaffolds can be significantly enhanced through modulating the concentration, modifications as well as designing GAGs analogues. In the future, endeavors should be taken to identify the key active domains of GAGs, and utilize their high affinity with ligands to improve the osteoconductive properties of materials in order to promote bone and cartilage regeneration.

## Future Challenges and Prospects

With the rapid development of glycomic and glycoproteomic approaches such as liquid chromatography tandem-mass spectrometry (LC-MS) (Bodet et al., 2017), GAGs microarray (Zong et al., 2017; Pomin and Wang, 2018), GAGome (Chen et al., 2018; Qiu et al., 2018), and bioinformatics, significant insights have been gained regarding the disaccharide composition, detailed structure of proteoglycans/GAGs, and their interactions with a wide range of biological molecules. Although these analyses have provided valuable insight into the biological functions of proteoglycans and GAGs, the most convincing approach to exploring their context-dependent functions lies in establishing specific animal models. Unfortunately, a large number of proteoglycans/GAGs-deficient animal models exhibit embryonic lethality. Future efforts should be taken to elucidate more detailed proteoglycans/GAGs functions with conditionally modified genetic models. In addition, understanding the functional overlap or compensation between proteoglycans/GAGs will also be important to defining the structure-function relationship of proteoglycans/GAGs. Endeavors should also be taken to improve our understanding of the distinct structural features of each proteoglycan subfamily and to recognizing the docking site for bio-active molecules within their core proteins and GAGs side chains.

## Concluding Remarks

Proteoglycans are ubiquitously expressed across tissues and exert a multitude of effects on stem cell behavior and the surrounding microenvironment. Accumulating evidence has highlighted the significance of proteoglycans and their biosynthetic machinery in regulating stem cell and tissue homeostasis. In particular, the sulfate groups on GAGs give rise to the complex structural heterogeneity of proteoglycans, and are essential for the interactions with growth factors in the regulation of tissue development and cell behavior. These properties support GAGs as valuable tools for bone tissue engineering as a bio-active scaffold or as an adjuvant for exogenous growth factor administration. With the advancements in biomimetic techniques, GAGs analogues designed to recapitulate the interactions between proteoglycans and growth factors, better outcomes are expected to be achieved in bone regeneration.
